# Quality improvement package for severe tuberculosis triage in Tamil Nadu, India

**DOI:** 10.5588/pha.26.0023

**Published:** 2026-08-24

**Authors:** A. Jeyakumar, S. Kalaiselvi, D. Nair, R. Vijayaprabha, K. Gayathri, D. Kabir, J.M. Melfha, T. Bhatnagar, R. Srinivasan, M. Balusamy, S Shanmugasundaram, Alicia Catherine, N Nithya, R Venkateshwar, A. Frederick, H.D. Shewade

**Affiliations:** 1 ICMR National Institute of Epidemiology (ICMR-NIE), Chennai, India;; 2 All India Institute of Medical Sciences (AIIMS) Madurai, India;; 3 ICMR National Institute for Research in Tuberculosis, Chennai, India;; 4 Directorate of Medical and Rural Health Services, Government of Tamil Nadu, Chennai, India.

**Keywords:** tuberculosis, triage, quality improvement, SORT IT, M-PHISOR

## Abstract

**SETTING:**

Tamil Nadu Kasanoi Erappila Thittam (TN-KET) is a statewide differentiated tuberculosis (TB) care initiative to reduce TB deaths involving triaging for severe illness at diagnosis and referral of severely ill for appropriate inpatient care (2022–2025). Eleven out of 30 districts did not reduce their TB death rates despite achieving the target process indicators.

**OBJECTIVE:**

To assess the implementation fidelity and effect of a quality improvement (QI) package to improve triage coverage and quality in the select 11 districts of Tamil Nadu.

**DESIGN:**

A quasi-experimental pre–post study was conducted between October 2024 to February 2025 (pre-QI) and June to December 2025 (post-QI). The QI package was introduced in poorly performing districts. Data were extracted from *Ni-kshay* and TB *SeWA* (e-tools of the TB programme and TN-KET, respectively), supplemented by independent triage audits.

**RESULTS:**

Integrating the QI package within TN-KET achieved high fidelity. The extent of missing triage data reduced from 38% to 12%, and median delays in entry of triage details reduced from 13 to 9 days and early TB deaths reduced from 3.7% to 3.3%.

**CONCLUSION:**

The QI package improved documentation and timeliness of triage assessment, contributing to modest reductions in early TB deaths. District-level supervision and triaging at diagnosis for early admission will sustain the reduced death rate.

Globally, tuberculosis (TB) remains a leading cause of mortality due to an infectious disease with an estimated 1.1 million TB-related deaths (referred to as TB deaths, henceforth) in 2024.^1,2^ India accounts for an estimated 2.7 million annual TB incidence and estimated 0.3 million annual TB deaths.^3^ To address preventable TB deaths, a differentiated TB care approach was introduced under India’s National TB Elimination Programme in January 2021.^3^

Tamil Nadu, a southern state of India, serves a population of around 80 million and has been recognised as one of the high-TB-burden states in the country. In 2022, approximately 88,600 people with TB were notified in Tamil Nadu, of whom 64,745 were diagnosed in public health facilities. Around 5,000 TB-related deaths were reported that year.^4^

In response, state-level discussions and prior experiences (including pilot use of a simple triage tool in Karnataka and Gujarat, as well as feasibility assessments of differentiated TB care guidance introduced in January 2021) helped to shape programmatic strategies.^5^

Building on these efforts, the Tamil Nadu Kasanoi Erappila Thittam (TN-KET, meaning ‘TB death–free initiative’) was launched in April 2022 across the state, covering all districts except Chennai. Under this initiative, all adults (≥15 years) diagnosed with TB (not known to be drug-resistant at diagnosis) in public health facilities were triaged at diagnosis for very severe undernutrition (body mass index ≤14 kg/m^2^ or 14.1–16 kg/m^2^ with bilateral pedal oedema), respiratory insufficiency (at rest, respiratory rate > 24 per minute or oxygen saturation < 94%), and poor performance status (unable to stand without support). Triage-positive adults (any one of the three conditions) were prioritised for comprehensive clinical assessment and inpatient care.^1,6,7^

This initiative was proposed to achieve a 30% relative reduction in TB deaths, and a decline in TB death rates was observed in several districts following its implementation. Despite achieving programme-reported triage coverage targets (90%), 11 out of 30 districts did not achieve the expected reduction in TB deaths. To identify the gaps in triage coverage and quality, we performed triage audit during October 2024 to February 2025. This triage audit identified that the effective triage coverage (based on individual-level tracking) was low compared to programme-reported triage coverage (based on aggregate numbers).^8^ It was also found that the extent of triage-positive cases (severely ill) reported from health facilities was lower compared to the audit. Gaps in failure to assess pedal oedema, inaccuracies in weight and body mass index measurement, and misclassification of triage status were also identified.^8^ These gaps could have resulted in severely ill patients not being identified and not receiving appropriate inpatient care, thereby contributing to preventable TB deaths.

Quality improvement (QI) approaches in TB care cascade are widely reported.^9–11^ QI is widely recognised as ‘a systems-based approach that addresses the “know-do” gap through a structured continuous process’.^10^ Addressing these implementation gaps through structured QI interventions was considered essential to improve triage coverage and quality and to strengthen the differentiated TB care cascade. In this context, QI package related to uptake of training, reach of resource materials, supportive supervision, actual performance of triage in the field, and tracking for triage based on the missing list generated was implemented. This study aimed to document the implementation and effect of this QI on effective triage coverage, quality, and early Tb deaths in public health facilities of the 11 districts in Tamil Nadu.

## METHODS

This was a quasi-experimental pre- and post-QI study nested within the differentiated TB care initiative model (TN-KET) implemented in routine programme settings of Tamil Nadu.

### Setting

Implemented between April 2022 and December 2025, TN-KET is India’s first statewide differentiated TB care initiative aimed at reducing TB deaths. A paper-based triage tool was introduced in all the public health facilities that diagnosed TB. A paper-based case record form was introduced in all the nodal inpatient care facilities in the district that managed triage-positive adults with TB. Key variables from the paper-based tools were entered in severe TB web application (TB *SeWA*).^7^ While TB *SeWA* was the e-tool of TN-KET, *Ni-kshay* is the web-based and case-based TB information management system of India’s NTEP. Pre-QI: As a part of the baseline audit of triage, gaps were identified during October 2024 to February 2025. The state technical support team independently assessed triage-related items and compared with the initial triage assessment among 158 people with TB (PwTB). Findings of this assessment are published already.^8^ From January 2026 onwards, Tamil Nadu shifted from TB *SeWA* to the differentiated TB care module in *Ni-kshay* which is in line with the revised March 2025 differentiated TB care guidance of NTEP.^4^

### Developing the change solution package

The QI audit cycle implemented under this study in 11 districts included the steps of identifying the problem, conducting root cause analysis, brainstorming solutions, developing a package of change solutions and an action plan, implementing the change in the field, reviewing the change measure, and deciding whether to continue or explore alternate change ideas.

Following the problem identification on triage coverage and triage quality, a root cause analysis was performed to identify the cause for these gaps. Accordingly, a conceptual framework was developed to indicate the resources and activities required to achieve the outcome (Supplementary Data Figure S1). The process from diagnosis to entry of triage details in TB *SeWA* was mapped to identify the major process bottlenecks. Similarly, the lack of skills and confidence among health care workers assigned for triaging of TB to record pedal oedema and respiratory rate was also expressed from the field. For these identified reasons, change solutions were explored and the draft package of action points identified were shared with state TB cell for consensus. Conceptual framework to guide the resources and activities for QI is illustrated in Supplementary Data Figure S1.

### Intervention package and implementation process

The QI package implemented to improve the quality of triaging is described in the Box.

BOX. Quality improvement (QI) package introduced under TN-KET to improve the coverage and quality of triaging in selected district of Tamil Nadu, India.

Refresher Training: A refresher training for NTEP staff including NTEP staff members, Medical Officers, and District TB Officers was conducted by the TSU members at the district level.

Access to Resource Materials (Job Aids): Job Aids were developed in Tamil language and distributed to illustrate the steps to be performed for triage among adults with TB. Videos on methods to assess triage-related parameters such as measurement of height and weight, respiratory rate, pedal oedema, and performance status were widely circulated in the professional WhatsApp group for all districts in Tamil Nadu.

District-Led Supportive Supervision: District programme managers were requested to review the triage coverage tracked from individual *Ni-kshay* ID and triage quality during their monthly review meeting.

Monitoring Triaging Quality: During the supervisory visits by the TSU team, the triage-positive and triage-negative adults with TB were assessed. Using the structured check list, health care providers were asked to demonstrate the triage items.

Real-Time Data Entry: The health staff were oriented on the relevance of timely entry of triage details under TB *SeWA* by the District programme managers. Monthly feedback on the percentage of adults with TB missed from triage and the duration of delay from triage to data entry was provided by the TSU to reflect the progress.

Tracking Non-Triaged Adult Drug-Sensitive PwTB Patients: Monthly *Ni-kshay* data were cross-checked against TB *SeWA* records, and the line list of PwTB who did not undergo triage was sent to programme managers and staff for triaging. TB unit–level staff were trained to extract the list of missing *Ni-kshay* IDs in TB *SeWA* and then track them for triage.

TB = tuberculosis

NTEP = National Tuberculosis Elimination Programme

TSU = Technical Support Unit

*Ni-kshay* = NI-KSHAY (Ni = End, Kshay = TB)

TB *SeWA* = Severe TB Web Application

PwTB = People with TB

### Study population

All adults (aged ≥ 15 years) with TB (not known to be drug-resistant at diagnosis) notified from public facilities in the 11 selected districts of Tamil Nadu between July–September 2024 and July–September 2025 were included. The health care staff involved in TB care and reporting in these districts were also included. Around 4,000 adults with TB were included to assess changes in triage coverage, delay in capture of triaging details in TB *SeWA* post-diagnosis, and early TB deaths (within 2 months of diagnosis). For triage quality assessment, a minimum of five triage-positive and 5% of triage-negative patients were included per district except one district where the notification was low. At least one adult with TB per public facility was randomly shadowed to observe triage practices.

### Data collection

Data on TB notification and early TB deaths were extracted from *Ni-kshay*. Data on triage coverage and delay in entry of triage details were extracted from TB *SeWA*. Independent triage assessments were conducted using structured checklists among recently diagnosed patients within 2 weeks of treatment. If recent records were unavailable in TB *SeWA*, paper-based triage tools were obtained from districts. Fidelity of QI package implementation was assessed through structured non-participant observations by audit team. Components included in the QI package and their corresponding fidelity indicators, along with data sources, are described in the Supplementary Data Table.

### Data management and analysis

List of eligible notified PwTB for July–September 2024 and July–September 2025 was extracted from Ni-kshay, and using this unique ID the status on triage was verified in the TB SeWA web portal. This is referred to as effective triage coverage. Median delays in triaging pre- and post-QI were compared using non-parametric tests. The proportion of early TB deaths were compared using parametric tests. Triage quality was assessed by comparing the TB *SeWA* records and independent assessment by audit team. Agreement and errors in measurement and reporting of triage indicators were calculated as proportions, and changes were compared. Implementation fidelity indicators, including training coverage, supportive supervision, and field-level triage practices, were summarised using frequencies and percentages.

### Ethical statement

The ethical approval was obtained from the Institute Human Ethics Committee of ICMR National Institute of Epidemiology (ICMR-NIE), Chennai, India. The informed consent was obtained from the patients and other health system stakeholders participating in the study. All necessary administrative approvals for implementing this triage audit planned under TN-KET were obtained from the state TB office.

## RESULTS

The QI package was implemented in all 11 districts. Training, dissemination of resource materials, and review of triage status happened in all districts. Onsite supportive supervision by district-level programme managers, tracking the list of persons with TB that were not triaged, and ensuring timely triage entry were not reported in all districts. Documentation of pedal oedema was observed in 65% (Table 1).

**TABLE 1.** Implementation fidelity^A^ of the quality improvement (QI) package for improving triage coverage and quality in selected 11 districts in Tamil Nadu, June–December 2025.

Indicator	N (%)
The quality of items assessed for triage among adults with tuberculosis (TB)	215 (100)
Weight measured at the time of diagnosis	205 (95)
SPO_2_ measured during triage	177 (82)
Assessed for pedal oedema	140 (65)
Adoption of the QI package at district level	11 (100)
Health care providers refresher training on triaging in the district	11 (100)
TB diagnostic facilities having display of job aids and demonstration videos on TB triage	10 (91)
Supportive supervision through onsite visit triage monitoring	5 (45)
TB units received list of non-triaged TB adults	11 (100)
Reviewed timeliness of data entry	9 (82)
Tracked the missed triage list shared	6 (55)
Districts reviewed the triage status in the monthly review	11 (100)
Skills to perform triage items	11 (100)
Weight measurement	9 (82)
Respiratory rate	8 (73)
Pedal oedema	8 (73)
Classification of severe TB	11 (100)

A Reach = % of person with TB got assessed for triage; adoption =– % of facilities initiated the implementation package.

### Outcome change observed in post-QI compared to pre-QI

#### Triage coverage and early TB deaths.

The effective triage coverage in 2024 was 87% (4,713/5,415) in 2024 and 84% (4,629/5,519) in 2025. Early TB deaths reduced from 3.7% to 3.3%.

#### Reporting

The data documentation improved, with reduced missing data on triage variables (38% to 12%) and reduced delays in entry of triage details (median 13 days to 9 days).

#### Triage quality

As shown in Table 2, there was an improvement in the documentation of respiratory rate and agreement in triage-positive status (pre-QI: 61% vs. post-QI: 81%) in the post-QI period compared to pre-QI.

**TABLE 2.** Comparison of triage quality after implementation of the quality improvement package in selected 11 districts of Tamil Nadu, 2024–2025.

Concordance in triage for severe tuberculosis (TB) status triage indicator	Pre: October 2024–February 2025 (N = 158)		Post: June–December 2025 (N = 215)	
	N	n (%)^A^	N	n (%)
Missing data on all triage items	158	60 (38)	215	25 (12)
Very severe undernutrition	91	77 (85)	178	149 (84)
Respiratory insufficiency (SPO_2_)	98	84 (86)	180	161 (89)
Respiratory insufficiency (respiratory rate)	–	–	176	104 (59)
Poor performance status	98	84 (86)	190	156 (82)
Triage-positive status	98	60 (61)	190	153 (81)

A % agreement for assessment between audit team and health system staff on triage indicators.

## DISCUSSION

The adoption of the QI package introduced under TN-KET-differentiated TB care model at public facilities in selected districts of Tamil Nadu showed varying implementation. All districts adopted activities related to imparting skills and resources to facilitate tracking, whereas the onsite supportive supervision and tracking for missed triage patients could not be adopted by all. At patient level, assessment of triage items which were neglected earlier such as pedal oedema assessment improved following QI. Though the effective triage coverage did not increase and early TB deaths increased modestly, the introduction of QI was associated with improved data quality in the form of reducing missing data and delays in data entry. The triage quality also improved post-QI period reflected as high level of agreement in identifying triage-positive between audit team and assessment made by the health staff similar to other studies.^9–12^

The inconsistent adoption of field-level supportive supervision and tracking for the missed people from triage indicates a major bottleneck at the health system level, a systemic challenge reported in contexts of other diseases as well.^13–15^ Our earlier published study discussed that the triage coverage based on aggregate reported numbers could overestimate the coverage with potential beneficiaries being left out.^8^ The reason for no improvement in effective triage coverage despite emphasis on timely documentation and targeted tracking could be partially explained by the programmatic decision to shift the entries in the differentiated TB care module of *Ni-kshay*. Though this happened from January 2026, the discussion to shift started at state level from October 2025. Hence, the changes in indicators reported by us are conservative.

The modest decrease in early TB deaths, despite greater adoption of capacity-building and technical resources for targeted tracking, underscores the importance of onsite supportive supervision and issuing directives to peripheral health facilities for targeted tracking. These factors, which have further scope for improvement, can act more proximally compared to other elements.^4^

As the QI package was nested in the ongoing TN-KET in routine programme settings, the response rate and fidelity for proposed activities were high. As the state shifts to the differentiated TB care module of *Ni-kshay* (in line with the March 2025 revised differentiated TB care guidance), which has a comprehensive list of variables, experience from this QI can help to understand the domains which need more attention while implementing in the field.^15^

Future research should focus on demonstrating a sustainable supportive supervision model ensuring the standard of care for TB. The available opportunities under digital information systems, such as the missing triage list, should be leveraged to ensure targeted tracking and monitor the patients.

## CONCLUSION

The adoption of QI under TN-KET-differentiated TB care model was associated with improvement in the triage quality, timeliness, and completeness of data entry, though the effective triage coverage and early TB deaths had only modest desirable change. Ensuring consistent field-level supportive supervision and systematic tracking of patients missed during triage are critical areas that require strengthening. The learnings from this QI package implementation could be applied as the state steps up to the differentiated TB care module of *Ni-kshay* that has a higher number of variables being used for severity assessment.

## Supplementary Material
