# Supplementary material for: Quality improvement package for severe tuberculosis triage in Tamil Nadu, India
Source: Public Health Action. 2026 Aug 24;16(3):168–71. doi: 10.5588/pha.26.0023 (PMC13502725; doi:10.5588/pha.26.0023)
Supplement: Supplementary file 1 [file pha26-0023_supplementarydata1.pdf]

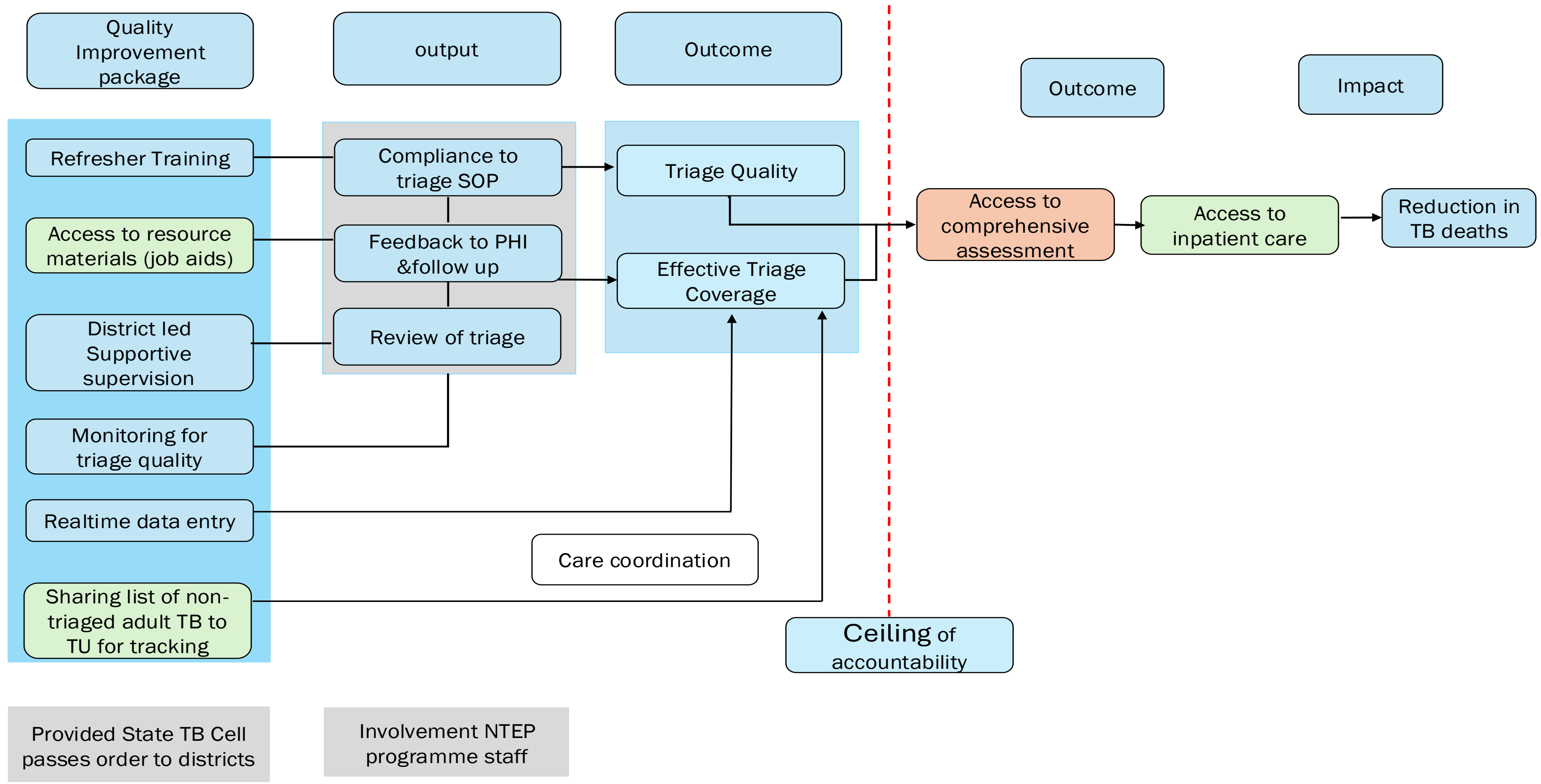

Supplementary Figure 1: Conceptual Framework to guide the resources and activities for Quality improvement

Supplementary Table 1. Items included under the QI package, fidelity indicators, and data sources under TN-KET 2025

Items under the QI package	Fidelity indicator	Specific domain addressed under the fidelity framework	Data source
Refresher training	% (districts) conducted reorientation training for health care workers involved in TB care	Coverage	Survey (among District TB officers)
Access to resource materials /job aids	% districts with TB diagnostic facilities having display of job aids and demonstration videos on TB triage<br><br>% of districts received the list of non-triaged TB adults from TSU	Coverage<br><br>Content	Survey<br><br>Survey
District-led supportive supervision	% of districts made a supportive supervision onsite visit for triage monitoring<br>% of district TB officers reviewed the triage status in the monthly review	Engagement (responsiveness)	Survey<br><br>Survey
Monitoring triage quality	% of PwTB assessed for items included under triage criteria<br><br>% of HCWs demonstrated appropriate skills to assess items included under triage	Adherence<br>Data documentation<br><br>Quality	Independent patient-level assessment by the audit team and comparison with the primary triage record<br><br>On-site non-participant observation
Real-time data entry	% of the district manager reviewed the timeliness of data entry	Documentation	Survey
Tracking non-triaged adult drug-sensitive TB	% of districts tracked the missed triage list shared from the TSU	Adherence	Survey
